# Association of time-varying sleep duration and cognitive function with mortality in the elderly: a 12-year community-based cohort study

**DOI:** 10.1186/s12888-023-05434-z

**Published:** 2023-12-20

**Authors:** Tsai-Chung Li, Chia-Ing Li, Chiu-Shong Liu, Chih-Hsueh Lin, Shing-Yu Yang, Cheng-Chieh Lin

**Affiliations:** 1 Department of Public Health, College of Public Health, China Medical University, Taichung, Taiwan; 2 Department of Healthcare Administration, College of Medical and Health Science, Asia University, Taichung, Taiwan; 3 School of Medicine, College of Medicine, China Medical University, No. 100, Sec. 1, Jingmao Rd., Beitun Dist., Taichung City, 406040 Taiwan ROC; 4 Department of Medical Research, China Medical University Hospital, Taichung, Taiwan; 5 Department of Family Medicine, China Medical University Hospital, Taichung, Taiwan

**Keywords:** Older adults, Mortality, Sleep duration, Mini-Mental State Examination

## Abstract

**Background:**

Sleeping problems and cognitive impairment are common in elders. Baseline sleep duration and cognitive status are predictors of mortality. But few studies have explored whether longitudinal changes in sleep duration and cognitive function are related to mortality in older adults. The present study investigated the time-varying relationships of sleep duration and cognitive function with subsequent mortality among community-dwelling elders by using 12 years of repeated-measure data.

**Methods:**

Taichung Community Health Study for Elders (TCHS-E) is a retrospective, population-based cohort that started in 2009 (wave 1) with a total of 912 elders aged 65 years or above. Follow up was conducted in 2010 (wave 2), 2018 (wave 3), and 2020 (wave 4). Sleep duration and Mini-Mental State Examination (MMSE) forms were executed at baseline and three visits during follow-up. Time-varying Cox proportional hazards regression estimated adjusted hazard ratios (HRs) of mortality with 95% confidence intervals (CIs).

**Results:**

During about 12 years (9,396 person-years) follow-up, 329 deaths from all causes were documented, including 102 deaths due to expanded cardiovascular disease (CVD). In the multivariable-adjusted, time-varying Cox proportional hazard model, the adjusted HR values of all-cause mortality were 1.47 (1.02–2.12) for sleep duration > 9 h/day (vs. 7 h/day) and 1.81 (1.26–2.59) for MMSE < 27 (vs. 30). The adjusted HR values of the expanded CVD mortality were 2.91 (1.24–6.83) for MMSE of 29; 2.69 (1.20–6.05) for MMSE of 27–28; and 4.32 (95% CI: 1.92–9.74) for MMSE < 27. The dose-dependent relationship was significant (*p* < 0.001). The combinations of sleep duration longer than 9 h/day and MMSE < 27 were linked with the highest risks for expanded CVD and all-cause mortality.

**Conclusions:**

Long sleep duration and low cognitive function were jointly and independently linked with higher risk of mortality in elders residing in community.

**Supplementary Information:**

The online version contains supplementary material available at 10.1186/s12888-023-05434-z.

## Background

The associations of sleep duration and cognitive function with subsequent adverse outcomes in elders have received increased research attention given that the world population is aging [1]. Sleeping problems and cognitive impairment are prevalent in the elders; in particular, about 50% of older adults have sleep problems [2] and more than 40% have cognitive impairment [3]. Sleeping is critical to maintain brain health because it can clear metabolic wastes from the brain [4] and support cognitive function and memory [5]. Sleep duration and cognitive loss individually lead to premature death in two meta‐analyses based on observational cohort studies [6, 7]. One of the meta-analysis identified 27 cohort studies and showed that long and short sleep durations were linked with increased risks of all-cause mortality in elders whereas long sleep duration was linked with cardiovascular mortality [6]. The other meta-analysis found 9 studies including 48,709 older individuals and reported that mild cognitive impairment was related to a 1.59-fold increase in mortality [7]. The interplay between sleep duration and cognitive function may confound their association with mortality; as such, the combined and independent effects of cognitive function and sleep duration on elders should be investigated for health monitoring.

A number of observational epidemiologic studies demonstrated the relationships of cognitive function and sleep duration with mortality from cross-sectional or cohort studies [8–17]. However, these studies did not consider simultaneously to explore their independent effects. In addition, limited data were reported on the dynamic associations of sleep duration and cognitive function with mortality [15–17]. Sleep duration and cognitive function undergo dynamic, time-varying changes throughout the lifespan of an individual [18, 19]. Previous studies chiefly focused on the adverse health effects of sleep duration and cognitive function measured at baseline [8–11, 15, 20]. Baseline measurement cannot determine whether the observed associations between exposures and outcomes are assessed by persistent exposure or whether decreased or increased exposure can change risk. After literature search, we found three recent studies conducted on elderly (aged ≥ 65 years) [15], postmenopausal women [16], and oldest-old (aged ≥ 80 years) [17] to examine the associations of time-varying sleep duration or cognitive function with mortality. One study including 10,768 persons aged ≥ 65 years found that sleep duration longer than 8 h per day, but not sleep duration shorter than 6 h per day, was associated with a 1.13-fold higher risk of all-cause mortality during a mean follow-up of 4.51 years. Another study consisting of 158,203 postmenopausal women aged 50–79 years was conducted to perform baseline and time-varying analyses; the results showed that sleep duration shorter than 5 h per day and longer than 9 per day were significantly associated with higher risk of total and cardiovascular disease (CVD) mortality [16]. The other study, which comprised 25,285 Chinese oldest-old adults aged ≥ 80 years, observed the persistent dose–response associations between cognitive impairment and mortality risk [17]. Thus far, longitudinal epidemiological investigations on time-varying cognitive function and sleep duration among elderly remain scarce [15–17]. The three cohort studies explored the impact of cognitive function or sleep duration without ruling out the confounding effects of the other factor. Furthermore, these studies are restricted to hospital sample, female gender, and special age groups in the oldest old. No study has evaluated the joint effect of time-varying sleep duration and cognitive function on death. Therefore, this study aims to assess the independent and combined associations of time-varying cognitive function and sleep duration with mortality during 12 years of follow up on community-dwelling elders.

## Methods

### Study design and subjects

The Taichung Community Health Study for Elders (TCHS-E) is a population-based prospective follow-up study carried on in 3,997 elderly aged 65 years and older and resided in North Area of Taichung, Taiwan in 2009. All elders of TCHS-E were identified from the population registry of Internal Affairs of Taichung City and invited to take part in by home visit, letter, and phone. There were a total of 2,750 eligible elders, and 1,347 of them were willing to participate, with an overall 49.0% response rate (2009). We excluded participants with missing information on sociodemographic factors (*n* = 42), lifestyle behavior (*n* = 9), history of disease (*n* = 356, including diabetes mellitus, hypertension, gout, hyperlipidemia, heart disease, arthritis, hyperuricemia, cancer, stroke, osteoporosis, cataract, sleep impairment, fall history, and taking sleeping pills), Mini-mental State Examination score (MMSE), frailty, or sleep duration (*n* = 28). Finally, 912 elderly participants were available for the analysis (Supplementary Fig. 1). They were followed up after 1, 10, and 12 years of baseline, resulting in 521 elders at wave 2 (2010), 218 elders at wave 3 (2018), and 97 elders in wave 4 data collection (2020) without considering those who were dead at subsequent years. All elders were required to undergo face-to-face interviews under a standardized questionnaire and check-up exams.

## Measurements

Data were collected using a standard procedure with standardized instruments. The standardized questionnaire consists of socio-demographic variables, lifestyle behaviors, frailty status, disease history, and its components, sleep duration, and cognitive function.

### Sociodemographic variables and lifestyle behaviors

Socio-demographic variables included gender, age, marital status and educational attainment. Lifestyle behavior included several variables, such as alcohol drinking, smoking habits, and leisure-time physical activity. Alcohol drinking was classified as never, current, and past. Those who self-reported they did not regularly drink wine, beer, or hard liquor were defined as never alcohol drinkers. Smoking was classified as never current, and past. Those who smoked < 100 cigarettes during their lifetime were never smokers; those who smoked at least 100 cigarettes but who did not currently smoke cigarettes were past smokers while who currently smoked as current smokers. Physical activity was classified as yes for those who exercised at least 30 min per week for at least 6 months. Weight (kilograms) was measured with an electronic medical scale (seca, Hamburg, Germany). Height (meters) was measured with a fixed stadiometer (seca). Body mass index (BMI) was derived as weight divided by height x height (kg/m^2^). Underweight was defined as BMI < 18.5, normal as BMI of 18.5–24.9 kg/m^2^, overweight as BMI of 25–29.9 kg/m^2^, and obesity as BMI > 30 kg/m^2^.

### Covariate variables

#### Disease history and medication use

All disease history and medication use were binary (yes, no). Disease history variables included diabetes mellitus, hypertension, gout, hyperlipidemia, heart disease, arthritis, hyperuricemia, cancer, stroke, osteoporosis, cataract, sleep disturbance, and fall history. Medication use variables included hyperlipidemia medications, hypertension medications, anti-diabetes medications, cardiovascular medications, and sleeping pills.

#### Frailty status

Frailty was determined based on the standardized, and well-established phenotype proposed by Fried et al. [21]. It consists of five components, namely, weakness, slowness, shrinking, low physical activity, and poor endurance and energy. Weakness was determined by grip strength when the participants were in the lowest baseline quintile according to groups of BMI and gender [21]. Slowness was determined by the slowest quintile of the population’s walking time according to groups of standing height and gender [21]. Shrinking was determined by weight loss (no intention) greater than 3 kg during the past year. Low physical activity was determined by a summative kilocalories expended score per week deriving from each person’s report regarding time spent on each type of physical activity per session, and frequency of physical activity per week. Poor endurance and energy was determined by two self-reported items regarding exhaustion from the Center for Epidemiological Studies-Depression Scale [22]. Persons with two or more above components were defined as frail, those with one or two components as pre-frail, and those with no component as robust.

#### Cognitive function

Cognitive function was a time-varying variable, which was assessed by the MMSE questionnaire at baseline, first and second followed waves of data collection. The MMSE questionnaire was developed by Folstein et al. [23] for grading assessment of persons with cognitive impairment. It has been commonly used as a screen tool in epidemiological studies for cognitive disorders and cognitive monitoring in clinical trials. It contains items about place (maximum score of 5) and time (maximum score of 5) orientation with a total of 10 points, calculation (arithmetic such as serial sevens) and attention (maximum score of 5), recall (maximum score of 3), registration (repeating lists of words, maximum score of 3), and language use (naming a watch and a pencil), basic motor skills (maximum score of 1), and comprehension (maximum score of 8). The total score ranged from 0 to 30. The cutoff point of 27 is based on quartiles, i.e., based on statistical criterion. This cutoff point 27 has been used to define mild cognitive impairment in literature [24, 25].

#### Sleep duration

Sleep duration was a time-varying variable, which was assessed at baseline, first and second followed waves of data collection on the same day as cognitive function. The cutoff point of sleep duration is based on quartiles. An interviewer collected data about lifestyle behaviors including sleep duration through standardized questionnaire at each wave of data collection. The questionnaires were collected by in-person interview with standardized questionnaire and the standardization process can minimize measurement errors. Sleep duration was determined by the item “What are typical hours of sleep in a 24-h period, including day-time naps?” The time frame was set at a week before the date of interview. This type of data collection approach assumes the respondent can accurately average his/her behavior into a single estimate. Although there was no data about the validity of this question, the information of this question was collected under good quality approach.

### Outcome ascertainment

The outcomes were all-cause mortality and expanded CVD mortality. Death information was ascertained through linkage with Health and Welfare Data Science Center database for cause of death data. The follow-up started with date of baseline and ended with the end of follow-up on December 31, 2021 or death. Deaths were grouped into all-cause and expanded CVD mortality (CVD: International Classification of Disease, Tenth Revision, Clinical Modification (ICD-10-CM) codes I00–I99, diabetes: ICD-10-CM codes E10– E14 and kidney diseases: ICD-10-CM N00–N29) [26].

### Statistical analysis

Descriptive statistics for the baseline characteristics were presented as number (proportion) and assessed by Chi-squared test or Fisher’s exact test for categorical variables. Continuous factors were presented as means ± standard deviations (SDs) and assessed by two-sample t test or analysis of variance (ANOVA). Time-varying Cox proportional hazards regression analyses were employed to evaluate the longitudinal associations of sleep duration and cognitive function with mortality by considering time-varying covariance when an independent variable changes over time during the 12-year follow-up period (baseline, first and second followed waves)*.* Hazard ratios (HRs) and 95% confidence intervals (CIs) were reported after multivariate adjustment [27]. The SAS codes followed the steps suggested by Thomas L [28]. The multivariate adjusted estimates of survival functions from the Cox models were applied for survival functions across groups of sleep duration and cognitive function. Tests for linear trend were performed by including sleep duration and MMSE quartiles as ordinal variables in the models. The dose‒response or non-linear relationship of sleep duration and MMSE to mortality was explored using restricted cubic spline plots based on multivariate Cox models.

Multivariate models were built for adjusting for the covariates by the following steps. In step 1, we built univariate models for sleep duration and cognitive function along with age, sex, and exercise program. In step 2, we built a multivariate model by simultaneously considering significant factors in the univariate models. In step 3, a multivariate model was additionally considered disease history and frailty status. All analyses were conducted with SAS version 9.4 (SAS, Cary, NC). A two-sided *p*-value < 0.05 was treated statistically significant.

## Results

At the end of the 12-year study period, 912 sleep duration and cognitive function assessments were made (9,396 person-years), and 329 deaths were recorded, of which 102 were attributed to expanded CVD. At baseline, the mean age was 74.03 years [SD = 6.48], 52.30% were male, and the median follow-up time was 12.04 years (interquartile range [IQR] 9.24–12.19). Table 1 shows the sociodemographic and clinical characteristics according to survival status and comparison. Statistically significant differences were observed in sex, age, marital status, BMI, physical activity, disease history of heart disease, hypertension, diabetes, cancer, stroke, cataract, and fall, taking sleep pills, and frailty status and its components.

**Table 1:** The comparisons of baseline socio-demographic factors, lifestyle behaviors, disease history, frailty status, sleep duration and cognitive function according to death status in older adults (*n* = 912)

Variables	Death N (%)		*p* value
	No (*N* = 583)	Yes (*N* = 329)	
***Socio-demographic factors***			
Men	272 (46.66)	205 (62.31)	< 0.001
Age (years)	72.08 ± 5.22	77.49 ± 7.03	< 0.001
65–74	413 (70.84)	120 (36.47)	
75–84	156 (26.76)	154 (46.81)	
> 85	14 (2.40)	55 (16.72)	
Education			0.57
No education	67 (11.49)	37 (11.25)	
Primary education	158 (27.10)	100 (30.40)	
Secondary or tertiary education	358 (61.41)	192 (58.36)	
Married	435 (74.61)	217 (65.96)	0.007
Body mass index (kg/m^2^)	24.56 ± 3.23	24.04 ± 3.92	0.04
< 18.5	13 (2.23)	24 (7.29)	
18.5–25	335 (57.46)	186 (56.53)	
25–30	203 (34.82)	102 (31.00)	
≥ 30	32 (5.49)	17 (5.17)	
***Lifestyle behaviors***			
Smoking	46 (7.89)	38 (11.55)	0.09
Alcohol drinking	79 (13.55)	39 (11.85)	0.53
Physical activity	448 (76.84)	212 (64.44)	< 0.001
***Disease history***			
Hypertension	270 (46.31)	198 (60.18)	< 0.001
Diabetes mellitus	74 (12.69)	77 (23.40)	< 0.001
Heart disease	142 (24.36)	133 (40.43)	< 0.001
Hyperlipidemia	157 (26.93)	72 (21.88)	0.11
Gout	58 (9.95)	44 (13.37)	0.14
Hyperuricemia	55 (9.43)	40 (12.16)	0.24
Arthritis	113 (19.38)	66 (20.06)	0.87
Osteoporosis	110 (18.87)	52 (15.81)	0.28
Stroke	17 (2.92)	39 (11.85)	< 0.001
Cataract	238 (40.82)	181 (55.02)	< 0.001
Cancer	23 (3.95)	29 (8.81)	0.004
Fall history	114 (19.55)	97 (29.48)	< 0.001
Sleep impairment	252 (43.22)	157 (47.72)	0.21
Taking sleeping pills	125 (21.44)	88 (26.75)	0.08
***Frailty status***			< 0.001
Robust (0 components)	297 (50.94)	72 (21.88)	
Pre-frailty (1 ~ 2 components)	259 (44.43)	184 (55.93)	
Frailty (≥ 3 component)	27 (4.63)	73 (22.19)	
***Frailty components***			
Shrinking	62 (10.63)	57 (17.33)	0.006
Poor endurance and energy	10 (1.72)	27 (8.21)	< 0.001
Low physical activity	84 (14.41)	81 (24.62)	< 0.001
Slowness	133 (22.81)	196 (59.57)	< 0.001
Weakness	116 (19.90)	157 (47.72)	< 0.001
***Sleep duration (h/day)***			
Wave 1	7.16 ± 1.64	7.67 ± 2.11	< 0.001
Wave 2	7.34 ± 1.46	7.78 ± 1.89	0.009
Wave 3	7.32 ± 1.47	8.16 ± 1.75	0.009
Wave 4	7.56 ± 1.85	7.75 ± 3.18	0.89
**MMSE score**			
Wave 1	27.79 ± 3.16	26.23 ± 4.39	< 0.001
Wave 2	27.67 ± 2.74	25.83 ± 4.55	< 0.001
Wave 3	28.24 ± 2.69	25.78 ± 4.21	0.01
Wave 4	27.04 ± 3.66	25.00 ± 2.83	0.44

*MMSE* Mini–Mental State Examination

Table 2 shows the sociodemographic and clinical characteristics stratified by categories of sleep duration and MMSE. Compared with older persons with sleep duration of 7 h/day, those who had > 9 h/day of sleep were more likely to be older, men, and pursuing secondary or tertiary education, were less likely to have physical activity, and had high prevalence of diabetes, stroke, cataract, sleep impairment, taking sleeping pills, frailty status, low physical activity, poor endurance and energy, weakness, and slowness. Those with MMSE score < 27 were more likely to be older, female, and less educated but were less likely to be married and physical active, have a history of psychiatric symptoms, heart disease, cataract, fall, sleep impairment, taking sleeping pills, and have frailty status, low physical activity, slowness, and weakness.

**Table 2:** The comparisons of baseline socio-demographic factors, lifestyle behaviors, disease history and frailty status according to sleep duration and cognitive function in older adults (*n* = 912)

	Sleep duration (h/day) N (%)					MMSE score N (%)				
Variables	< 7 (*N* = 234)	**7** (*N* = 225)	8–9 (*N* = 359)	> 9 (*N* = 94)	*p* value	30 (*N* = 253)	29 (*N* = 215)	27–28 (*N* = 220)	< 27 (*N* = 224)	*p* value
***Socio-demographic factors***										
Men	94 (40.17)	115 (51.11)	205 (57.10)	63 (67.02)	< 0.001	150 (59.29)	127 (59.07)	121 (55.00)	79 (35.27)	< 0.001
Age (years)	73.53 ± 5.55	73.08 ± 6.33	74.12 ± 6.66	77.20 ± 7.34	< 0.001	72.09 ± 5.62	72.91 ± 6.12	74.23 ± 6.22	77.09 ± 6.86	< 0.001
65–74	139 (59.4)	147 (65.33)	210 (58.5)	37 (39.36)		181 (71.54)	142 (66.05)	122 (55.45)	88 (39.29)	
75–84	87 (37.18)	67 (29.78)	117 (32.59)	39 (41.49)		64 (25.3)	64 (29.77)	84 (38.18)	98 (43.75)	
> 85	8 (3.42)	11 (4.89)	32 (8.91)	18 (19.15)		8 (3.16)	9 (4.19)	14 (6.36)	38 (16.96)	
Education					0.02					< 0.001
No education	40 (17.09)	25 (11.11)	27 (7.52)	12 (12.77)		1 (0.40)	4 (1.86)	14 (6.36)	85 (37.95)	
Primary education	66 (28.21)	63 (28)	108 (30.08)	21 (22.34)		54 (21.34)	54 (25.12)	73 (33.18)	77 (34.38)	
Secondary or tertiary education	128 (54.7)	137 (60.89)	224 (62.4)	61 (64.89)		198 (78.26)	157 (73.02)	133 (60.45)	62 (27.68)	
Married	163 (69.66)	160 (71.11)	264 (73.54)	65 (69.15)	0.71	204 (80.63)	174 (80.93)	157 (71.36)	117 (52.23)	< 0.001
Body mass index (kg/m^2^)	24.05 ± 3.81	24.65 ± 3.27	24.47 ± 3.46	24.13 ± 3.37	0.52	24.37 ± 3.15	24.36 ± 3.34	24.46 ± 3.61	24.29 ± 3.91	0.90
< 18.5	12 (5.13)	6 (2.67)	12 (3.34)	7 (7.45)		8 (3.16)	7 (3.26)	7 (3.18)	15 (6.7)	
18.5–25	142 (60.68)	125 (55.56)	205 (57.1)	49 (52.13)		142 (56.13)	129 (60)	129 (58.64)	121 (54.02)	
25–30	67 (28.63)	84 (37.33)	120 (33.43)	34 (36.17)		93 (36.76)	68 (31.63)	71 (32.27)	73 (32.59)	
≥ 30	13 (5.56)	10 (4.44)	22 (6.13)	4 (4.26)		10 (3.95)	11 (5.12)	13 (5.91)	15 (6.7)	
***Lifestyle behaviors***										
Smoking	18 (7.69)	20 (8.89)	38 (10.58)	8 (8.51)	0.67	22 (8.7)	25 (11.63)	18 (8.18)	19 (8.48)	0.57
Alcohol drinking	24 (10.26)	36 (16)	47 (13.09)	11 (11.7)	0.32	39 (15.42)	30 (13.95)	28 (12.73)	21 (9.38)	0.25
Physical activity	173 (73.93)	178 (79.11)	251 (69.92)	58 (61.7)	0.008	198 (78.26)	161 (74.88)	162 (73.64)	139 (62.05)	< 0.001
***Disease history***										
Hypertension	108 (46.15)	107 (47.56)	200 (55.71)	53 (56.38)	0.06	126 (49.8)	106 (49.3)	107 (48.64)	129 (57.59)	0.19
Diabetes mellitus	38 (16.24)	26 (11.56)	62 (17.27)	25 (26.6)	0.01	42 (16.6)	32 (14.88)	35 (15.91)	42 (18.75)	0.73
Heart disease	73 (31.2)	66 (29.33)	105 (29.25)	31 (32.98)	0.88	80 (31.62)	66 (30.7)	49 (22.27)	80 (35.71)	0.02
Hyperlipidemia	68 (29.06)	60 (26.67)	83 (23.12)	18 (19.15)	0.19	71 (28.06)	55 (25.58)	51 (23.18)	52 (23.21)	0.56
Gout	21 (8.97)	33 (14.67)	38 (10.58)	10 (10.64)	0.26	38 (15.02)	17 (7.91)	24 (10.91)	23 (10.27)	0.10
Hyperuricemia	21 (8.97)	24 (10.67)	38 (10.58)	12 (12.77)	0.78	28 (11.07)	16 (7.44)	21 (9.55)	30 (13.39)	0.22
Arthritis	53 (22.65)	42 (18.67)	71 (19.78)	13 (13.83)	0.32	47 (18.58)	39 (18.14)	45 (20.45)	48 (21.43)	0.79
Osteoporosis	49 (20.94)	48 (21.33)	56 (15.6)	9 (9.57)	0.03	41 (16.21)	32 (14.88)	37 (16.82)	52 (23.21)	0.10
Stroke	9 (3.85)	10 (4.44)	21 (5.85)	16 (17.02)	< 0.001	10 (3.95)	11 (5.12)	14 (6.36)	21 (9.38)	0.09
Cataract	125 (53.42)	86 (38.22)	160 (44.57)	48 (51.06)	0.008	96 (37.94)	96 (44.65)	95 (43.18)	132 (58.93)	< 0.001
Cancer	7 (2.99)	17 (7.56)	20 (5.57)	8 (8.51)	0.11	15 (5.93)	14 (6.51)	12 (5.45)	11 (4.91)	0.90
Fall history	64 (27.35)	42 (18.67)	79 (22.01)	26 (27.66)	0.10	38 (15.02)	41 (19.07)	45 (20.45)	87 (38.84)	< 0.001
Sleep impairment	142 (60.68)	93 (41.33)	140 (39)	34 (36.17)	< 0.001	103 (40.71)	99 (46.05)	86 (39.09)	121 (54.02)	0.006
Taking sleeping pills	69 (29.49)	57 (25.33)	72 (20.06)	15 (15.96)	0.02	43 (17.00)	48 (22.33)	52 (23.64)	70 (31.25)	0.003
***Frailty status***					< 0.001					< 0.001
Robust (0 components)	87 (37.18)	106 (47.11)	148 (41.23)	28 (29.79)		134 (52.96)	98 (45.58)	85 (38.64)	52 (23.21)	
Pre-frailty (1 ~ 2 components)	131 (55.98)	110 (48.89)	165 (45.96)	37 (39.36)		106 (41.9)	105 (48.84)	113 (51.36)	119 (53.13)	
Frailty (≥ 3 component)	16 (6.84)	9 (4.00)	46 (12.81)	29 (30.85)		13 (5.14)	12 (5.58)	22 (10)	53 (23.66)	
***Frailty components***										
Shrinking	37 (15.81)	27 (12.00)	41 (11.42)	14 (14.89)	0.41	22 (8.7)	31 (14.42)	30 (13.64)	36 (16.07)	0.09
Poor endurance and energy	10 (4.27)	1 (0.44)	14 (3.9)	12 (12.77)	< 0.001	12 (4.74)	6 (2.79)	6 (2.73)	13 (5.8)	0.27
Low physical activity	35 (14.96)	26 (11.56)	79 (22.01)	25 (26.6)	0.001	39 (15.42)	34 (15.81)	35 (15.91)	57 (25.45)	0.01
Slowness	88 (37.61)	60 (26.67)	129 (35.93)	52 (55.32)	< 0.001	51 (20.16)	54 (25.12)	82 (37.27)	142 (63.39)	< 0.001
Weakness	60 (25.64)	55 (24.44)	108 (30.08)	50 (53.19)	< 0.001	47 (18.58)	56 (26.05)	65 (29.55)	105 (46.88)	< 0.001

Table 3 presents the HRs of time-varying Cox regression for all-cause and expanded CVD mortality. Time-varying sleep duration and MMSE score were associated with a high risk of all-cause mortality (HR: 1.76, 95%CI: 1.25–2.48 for sleep duration > 9 h/day, p for trend = 0.006; 2.00, 1.43–2.81 for MMSE score < 27, *p* for trend < 0.001) and expanded CVD mortality (2.04, 1.12–3.69 for sleep duration > 9 h/day, p for trend = 0.005; and 2.49, 1.09–5.70 for MMSE score of 29, 2.77, 1.26–6.09 for MMSE score of 27–28, and 4.21, 1.93–9.18 for MMSE score ≤ 26, *p* for trend < 0.001) when only adjusted for age, sex, and exercise program. After controlling for sociodemographic factors and lifestyle behaviours, the magnitude of associations of time-varying sleep duration and MMSE score was attenuated but remained statistically significant for all-cause mortality (1.74, 1.23–2.47 for sleep duration > 9 h/day, p for trend = 0.01; and 1.97, 1.37–2.82 for MMSE score ≤ 26, *p* for trend < 0.001) and expanded CVD mortality (2.13, 1.17–3.90 for sleep duration > 9 h/day, p for trend = 0.005; and 2.43, 1.06–5.56 for MMSE score of 29, 2.51, 1.14–5.54 for MMSE score of 27–28, and 4.27, 1.91–9.57 for MMSE score ≤ 26, *p* for trend < 0.001). Finally, in the fully adjusted model, the relationships of time-varying sleep duration and MMSE score with all-cause mortality remained statistically significant (1.47, 95%CI: 1.02–2.12 for sleep duration > 9 h/day, p for trend = 0.10 and 1.81, 1.26–2.59 for MMSE score < 27, *p* for trend = 0.002). Meanwhile, time-varying MMSE score, but not sleep duration, were significantly and dose-dependently associated with expanded CVD mortality (2.91, 1.24–6.83 for MMSE score of 29, 2.69 1.20–6.05 for MMSE score of 27–28, 4.32, 1.92–9.74 for MMSE score < 27, *p* for trend < 0.001). In order to assess the robustness of the results, a sensitivity analysis was performed using the sleep duration and MMSE at baseline to predict mortality (shown in Supplementary Table 1). The results remain similar to those in Table 3 although the attenuation of HRs for MMSE and sleep duration was observed.

**Table 3:** Time-varying Cox regression analysis for sleep duration and MMSE on mortality in older adults (*n* = 912)

Variables	N	Cases	Person-years	IR	HR (95%CI)					
					All-cause mortality			Expanded CVD mortality		
					Model 1	Model 2	Model 3	Model 1	Model 2	Model 3
**Sleep duration (hrs/day)**										
< 7	234	70	2546	27.49	0.99 (0.71, 1.39)	1.03 (0.73, 1.44)	1.04 (0.74, 1.47)	0.78 (0.41, 1.47)	0.78 (0.41, 1.50)	0.97 (0.50, 1.91)
7	225	72	2387	30.16	1.00	1.00	1.00	1.00	1.00	1.00
8–9	359	134	3657	36.65	1.10 (0.82, 1.48)	1.09 (0.81, 1.47)	1.07 (0.79, 1.45)	1.12 (0.65, 1.91)	1.09 (0.63, 1.86)	1.14 (0.65, 2.00)
> 9	94	53	805	65.82	1.76 (1.25, 2.48)**	1.74 (1.23, 2.47)**	1.47 (1.02, 2.12)*	2.04 (1.12, 3.69)*	2.13 (1.17, 3.90)*	1.78 (0.93, 3.41)
**P for trend**					0.006	0.01	0.10	0.005	0.005	0.10
**MMSE score**										
30	253	65	2777	23.40	1.00	1.00	1.00	1.00		1.00
29	215	68	2307	29.48	1.25 (0.87, 1.81)	1.21 (0.84, 1.75)	1.22 (0.84, 1.77)	2.49 (1.09, 5.70)*	2.43 (1.06, 5.56)*	2.91 (1.24, 6.83)*
27–28	220	77	2273	33.87	1.31 (0.92, 1.85)	1.27 (0.90, 1.81)	1.30 (0.92, 1.85)	2.77 (1.26, 6.09)*	2.51 (1.14, 5.54)*	2.69 (1.20, 6.05)*
<27	224	119	2038	58.39	2.00 (1.43, 2.81)***	1.97 (1.37, 2.82)***	1.81 (1.26, 2.59)**	4.21 (1.93, 9.18)***	4.27 (1.91, 9.57)***	4.32 (1.92, 9.74)***
**P for trend**					< 0.001	< 0.001	0.002	< 0.001	< 0.001	< 0.001

^*^p < 0.05; **p < 0.01; ***p < 0.001

Model 1 adjusted for age, sex and exercising program

Model 2 adjusted for age, sex, exercising program, education, marital status, BMI, smoking, alcohol drinking and physical activity

Model 3 adjusted for age, sex, exercising program, education, marital status, BMI, smoking, alcohol drinking, physical activity, hypertension, diabetes mellitus, heart disease, hyperlipidemia, gout, hyperuricemia, arthritis, osteoporosis, stroke, cataract, cancer, fall history, sleep impairment, taking sleeping pills and frailty

Incidence rate (IR) = number of incident cases / person-years*1000; *HR* hazard ratio, *CI* confidence interval, *MMSE* Mini–Mental State Examination

**Fig. 1:**
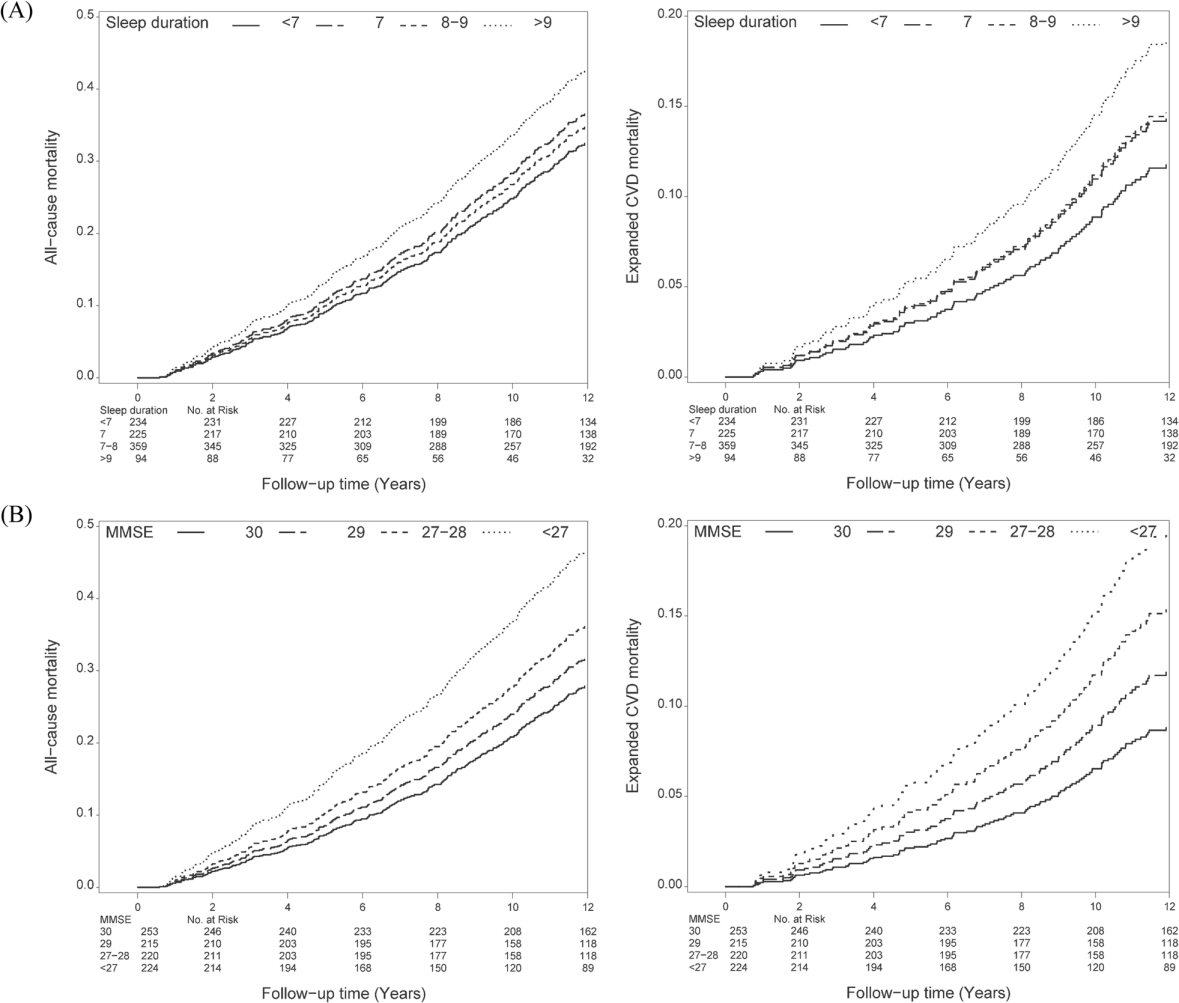
Multivariate adjusted survival curves of death for (**A**) sleep duration and (**B**) MMSE score

**Fig. 2:**
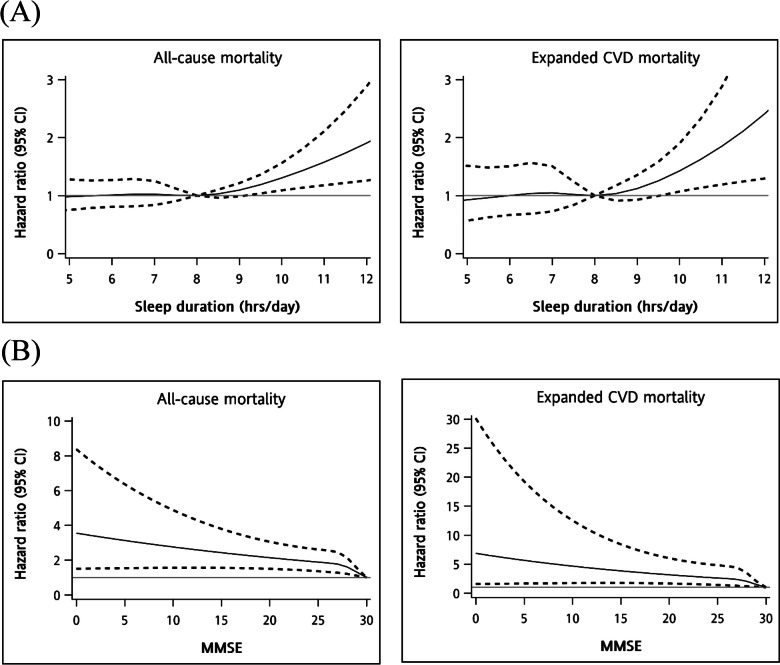
Multivariable cubic spline plots of (**A**) sleep duration and (**B**) MMSE score

The adjusted estimates of 12-year all-cause and expanded CVD mortality rates were 32.48% and 11.76% for sleep duration < 7 (h/day), 36.61% and 14.39% for sleep duration of 7 (h/day), 34.89% and 14.65% for sleep duration of 7–8 (h/day), and 42.59% and 18.69% for sleep duration > 9 (h/day), respectively; moreover, the values were 27.86% and 8.81% for MMSE score of 30; 31.68% and 11.90% for MMSE score of 29; 36.21% and 15.36% for MMSE score of 27–28; and 46.58% and 19.67% for MMSE score < 27, respectively (Fig. 1). We fitted the restricted cubic spline models to show the non-linear relationship between sleep duration **(**Fig. 2A) and MMSE score (Fig. 2B) for all-cause and expanded CVD mortality, respectively.

**Fig. 3:**
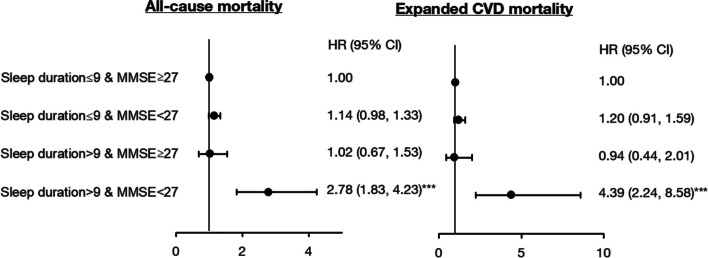
Joint effect of sleep duration and MMSE on risk for all-cause and expanded CVD mortality. Adjusted for age, sex, exercising program, education, marital status, BMI, smoking, alcohol drinking, physical activity, hypertension, diabetes mellitus, heart disease, hyperlipidemia, gout, hyperuricemia, arthritis, osteoporosis, stroke, cataract, cancer, fall history, sleep impairment, taking sleeping pills and frailty

Figure 3 presents the joint associations of sleep duration and MMSE with the risk of all-cause and expanded CVD mortality. In the joint effect models, older persons with longer sleep duration (> 9 h/day) and lower MMSE score (< 27) had the highest risk of all-cause and expanded CVD mortality (2.78, 1.83–4.23; 4.39, 2.24–8.58, respectively) compared with those with sleep duration ≤ 9 h/day and high MMSE score (≥ 27) while individual long sleep duration (> 9 h/day) and low MMSE score did not affect all-cause and expanded CVD mortality. In addition, the interaction terms for sleep duration and MMSE were significant for both all-cause and expanded CVD mortality (*p* = 0.01 and *p* = 0.02, respectively).

## Discussion

This study demonstrates that time-varying sleep duration and cognitive function can independently predict mortality by ruling out the confounding effects of the other factor among community-dwelling elders in a 12-year follow-up period (2009–2020). Older people with long sleep duration (> 9 h/day) or the lowest MMSE quartile (< 27) had 47% and 81% higher risks of death, respectively; meanwhile, those who had MMSE scores of 29, 27–28, and > 27 had 2.91-fold, 2.69-fold, and 4.32-fold increases in the risk of expanded CVD mortality compared with older persons with MMSE score of 30. A significant dose-dependent relationship was found between time-varying MMSE and expanded CVD mortality but not between sleep duration and expanded CVD mortality. The joint effects of time-varying sleep duration and cognitive function were significantly linked with the risk of all-cause and expanded CVD mortality. These findings well support that time-varying sleep duration and MMSE score jointly influence death.

The joint association of dynamic changes in cognitive function and sleep duration over time with mortality was first reported in elders residing in community. Our study found 2.78- and 4.39-fold increases in the risk of all-cause and expanded CVD mortality, respectively, in elders with sleep duration greater than 9 h/day and MMSE score < 27. Previous studies that used time-varying analyses showed a dose–response relation between MMSE score and mortality [17], consistent with the present report. The findings regarding the relationships between sleep duration and mortality are conflicting. Our study found an elevated risk of mortality in elders with sleep duration longer than 9 h/day. A previous study showed a J-shaped relation between sleep duration and mortality [15]. Another study on postmenopausal women described the U-shaped relationships of sleep duration with mortality in time-varying analyses [16]. The discrepancy in the results could be attributed to two reasons. First, gender may modify the relationships between sleep duration and mortality, and the difference increased with follow-up time [29]. Therefore, the estimated relationships reported in postmenopausal women may not be true in the elderly population. Second, the distribution of sleep duration varied in these studies; as such, they may not have sufficient power to detect the relationships between sleep duration less than 7 h and mortality. However, these studies, including the present work, found that sleep duration longer than 9 h was linked with mortality [15, 16].

This line of research question is biologically plausible and can be supported by several biological mechanisms. The autonomic nervous system may play a crucial role in the human body and controls many important physiological functions, including sleep initiation, maintenance, and disruption [30]. Sleep problems, such as long or short duration, and sleep disorders, including sleep apnea, insomnia, and a decrease in sleep quality resulting from nap during day time, are associated with a series of adverse cognitive and health outcomes, which may be explained by the outcomes of autonomic dysfunction due to sleep-associated alterations [31, 32]. Sleep disturbances may be associated with autonomic dysfunction [33] and metabolic disorders, resulting in obesity [34], hypertension [35], and insulin resistance [35]. Insulin in the brain plays a modulatory role for learning and memory [36]. Animal and clinical studies indicate that insulin uptake from blood into the brain is crucial for cognition through neuronal signaling [37–40]. Brain insulin resistance may cause cognitive impairment and cerebrovascular lesions [37, 38]. This impairment process is associated with the misfolding and extracellular aggregation of amyloid-β (Aβ) peptides [41] and hyperphosphorylated tau [42] because of the inadequate function of the system to clear these toxic substances in the brain. The outcomes of sleep disturbances include the following: disorders in endocrine [43], impairment of immune function [43], oxidative stress [44], increase in inflammatory reactions [45], and endothelial dysfunction [44, 46], and impaired physiology/diseases [47–49], including obesity, hypertension, diabetes mellitus, stroke, and depression. These phenomena may cause adverse outcomes, such as white matter hyperintensity[50], cerebrovascular abnormality [51], cardiovascular death [52], and total death [53].

The major strengths of our study include the following: a longitudinal cohort targeting community-dwelling elders with more than 12 years of follow up, standardized protocols and instruments for subject recruitment and data collection, and controlling for a large umber of potential confounding variables, such as educational attainment, sleep impairment, taking sleeping pills, frailty status, etc. In addition, this study used time-varying sleep duration and MMSE scores in the follow-up period and accurately assessed the associations by reducing misclassification in sleep duration and MMSE scores to a large extent. Nevertheless, this study has the following limitations. First, a previous study pointed out short sleep duration was linked with an increased risk of all-cause mortality among elderly [6]. However, our sample size is not large enough or the outcome events in short sleep duration are few to detect significant findings. Second, sleep duration data are derived from self-reports, which might lead to measurement error. By contrast, studies found that self-report sleep was well-correlated with objective sleep measurement, such as wrist actigraphy [54, 55]. Third, although adjustment was conducted for many confounders, the influence of unrecognized and residual confounding factors may exist because our study is observational. Fourth, the attrition rate is high because it is difficult to follow up a cohort of elders for more than 12-year period. Thus, only about 30% of baseline older adults had three measurements (baseline, first and second followed-waves) over a 12-year period. Fifth, in order to assess the potential selection bias arising from the differences between those who had been excluded and included, we made comparisons in baseline characteristics between them (shown in Supplementary Table 2). Those who were excluded tended to be female gender, older, with a lower educational level, non-married, obese, with a lower level of physical activity, and to have a higher prevalence of hypertension, diabetes, heart disease, gout, hyperuricemia, stroke, cataract, fall history, and frailty, but to have a lower prevalence of cancer compared with those who were included. If there exists an association between cognitive function and mortality, the present study would underestimate the association between cognitive function and mortality because these excluded persons were more likely to have cognitive impairment. The underestimation of effect results in the effect toward the null, a lesser threat to validity. Last, because the measurement of sleep duration was determined by the item “What are typical hours of sleep in a 24-h period, including day-time naps?” We didn’t have a question separately asking about day-time naps information. Thus, we cannot explore the association between day-time naps and cognitive function and mortality.

## Conclusion

Among older individuals, time-varying sleep duration and MMSE score are linked with increased risks of all-cause mortality, while time-varying MMSE score is associated with increased risks of expanded CVD mortality. Our study’s findings suggest time-varying sleep duration and MMSE score could be used as predictors of long-term death risk. As such, they should be incorporated into interventions for healthy sleep habits to improve survival and reduce immature mortality.

### Supplementary Information

**Additional file 1: Supplementary Fig. 1.** The flowchart of recruitment procedures of the current study. **Supplementary Table 1.** Cox regression analysis for sleep duration and MMSE on mortality in older adults (*n*=912). **Supplementary Table 2.** The comparisons of baseline socio-demographic factors, lifestyle behaviors, disease history, frailty status, sleep duration and cognitive function between the subjects excluded and included.

## Data Availability

The datasets generated and/or analyzed during the current study are not publicly available due to the policy declared by Ministry of Health and Welfare in Taiwan but are available from the corresponding author on reasonable request.

## Abbreviations

CVD: Cardiovascular disease
TCHS-E: Taichung Community Health Study for Elders
MMSE: Mini-Mental State Examination
BMI: Body mass index
ICD-10-CM: International Classification of Disease, Tenth Revision, Clinical Modification
SDs: Standard deviations
ANOVA: Analysis of variance
HRs: Hazard ratios
CIs: Confidence intervals
IQR: Interquartile range
