# Supplementary material for: Association of time-varying sleep duration and cognitive function with mortality in the elderly: a 12-year community-based cohort study
Source: BMC Psychiatry. 2023 Dec 20;23:954. doi: 10.1186/s12888-023-05434-z (PMC10731683; doi:10.1186/s12888-023-05434-z)
Supplement: Supplementary file 1 — Additional file 1: Supplementary Fig. 1. The flowchart of recruitment procedures of the current study. Supplementary Table 1. Cox regression analysis for sleep duration and MMSE on mortality in older adults (n=912). Supplementary Table 2. The comparisons of baseline socio-demographic factors, lifestyle behaviors, disease history, frailty status, sleep duration and cognitive function between the subjects excluded and included. [file 12888_2023_5434_MOESM1_ESM.docx]

2,750 eligible subjects

Eight administrative neighborhoods units in Taichung community

3,997 individuals with age ≥ 65 years old

1,247 excluded

122 deaths

124 errors of the registry

52 institutionalization

949 moving out of the area

886 refused to participate

490 not met at home during three visits made by the interviewers

27 hospitalization

1,347 elders agreed to participate

(response rate = 49.0%)

912 elders were included for analysis

42 without sociodemographic factors

9 without lifestyle behaviors

356 without disease history

28 without frailty, MMSE or sleep duration

**Supplementary Fig. 1.** The flowchart of recruitment procedures of the current study

**Supplementary Table 1.** Cox regression analysis for sleep duration and MMSE on mortality in older adults (n=912)

|  | HR (95%CI) | | | | | |
| --- | --- | --- | --- | --- | --- | --- |
|  | All-cause mortality | | | Expanded CVD mortality | | |
| Variables | Model 1 | Model 2 | Model 3 | Model 1 | Model 2 | Model 3 |
| ***Sleep duration (hrs/day)*** | |  |  |  |  |  |
| <7 | 0.88 (0.63- 1.22) | 0.87 (0.62- 1.21) | 0.83 (0.59- 1.18) | 0.77 (0.40- 1.46) | 0.78 (0.40- 1.49) | 0.77 (0.39- 1.51) |
| 7 | 1.00 | 1.00 | 1.00 | 1.00 | 1.00 | 1.00 |
| 8-9 | 1.10 (0.83- 1.47) | 1.05 (0.78- 1.40) | 0.91 (0.67- 1.23) | 1.38 (0.81- 2.35) | 1.26 (0.74- 2.16) | 1.03 (0.58- 1.81) |
| >9 | 1.62 (1.13- 2.33)** | 1.57 (1.09- 2.27)* | 1.29 (0.88- 1.90) | 1.99 (1.05- 3.78)* | 2.00 (1.04- 3.84)* | 1.44 (0.72- 2.89) |
| **P for trend** | 0.004 | 0.007 | 0.10 | 0.003 | 0.006 | 0.11 |
| **MMSE score** |  |  |  |  |  |  |
| 30 | 1.00 | 1.00 | 1.00 | 1.00 |  | 1.00 |
| 29 | 1.16 (0.82- 1.63) | 1.14 (0.81- 1.61) | 1.19 (0.84- 1.69) | 1.19 (0.61- 2.34) | 1.15 (0.58- 2.26) | 1.45 (0.72- 2.93) |
| 27-28 | 1.30 (0.93- 1.82) | 1.27 (0.91- 1.79) | 1.42 (1.01- 1.99)* | 1.66 (0.88- 3.12) | 1.61 (0.85- 3.04) | 2.00 (1.04- 3.85)* |
| <27 | 2.37 (1.71- 3.28)*** | 2.42 (1.70- 3.44)*** | 2.18 (1.53- 3.11)*** | 2.91 (1.58- 5.39)*** | 2.87 (1.50- 5.52)** | 2.87 (1.49- 5.53)** |
| **P for trend** | <0.001 | <0.001 | <0.001 | <0.001 | <0.001 | <0.001 |

*:p<0.05; **:p<0.01; ***:p<0.001.

Model 1 adjusted for age, sex and exercising program.

Model 2 adjusted for age, sex, exercising program, education, marital status, BMI, smoking, alcohol drinking and physical activity.

Model 3 adjusted for age, sex, exercising program, education, marital status, BMI, smoking, alcohol drinking, physical activity, hypertension, diabetes mellitus, heart disease, hyperlipidemia, gout, hyperuricemia, arthritis, osteoporosis, stroke, cataract, cancer, fall history, sleep impairment, taking sleeping pills and frailty.

Incidence rate (IR) = number of incident cases / person-years*1000; HR: hazard ratio; CI: confidence interval, MMSE: Mini–Mental State Examination.

**Supplementary Table 2.** The comparisons of baseline socio-demographic factors, lifestyle behaviors, disease history, frailty status, sleep duration and cognitive function between the subjects excluded and included

|  | Subjects N (%) | | Standardized effect size |
| --- | --- | --- | --- |
| Variables | Excluded (N=435) | Included (N=912) |  |
| ***Socio-demographic factors*** |  |  |  |
| Men | 172 (39.54) | 477 (52.30) | -0.26 |
| Age (years) | 76.45±6.97 | 74.03±6.48 | 0.37 |
| 65-74 | 194 (44.6) | 533 (58.44) | -0.28 |
| 75-84 | 178 (40.92) | 310 (33.99) | 0.14 |
| >85 | 63 (14.48) | 69 (7.57) | 0.23 |
| Education |  |  | 0.57 |
| No education | 60 (13.82) | 104 (11.40) | 0.07 |
| Primary education | 170 (39.17) | 258 (28.29) | 0.23 |
| Secondary or tertiary education | 204 (47.00) | 550 (60.31) | -0.27 |
| Married | 261 (61.85) | 652 (71.49) | -0.21 |
| Body mass index (kg/m^2^) | 24.66±3.83 | 24.37±3.50 | 0.08 |
| <18.5 | 16 (3.96) | 37 (4.06) | -0.01 |
| 18.5-25 | 217 (53.71) | 521 (57.13) | -0.07 |
| 25-30 | 136 (33.66) | 305 (33.44) | 0.00 |
| ≥30 | 35 (8.66) | 49 (5.37) | 0.13 |
| ***Lifestyle behaviors*** |  |  |  |
| Smoking | 37 (8.73) | 84 (9.21) | -0.02 |
| Alcohol drinking | 56 (13.21) | 118 (12.94) | 0.01 |
| Physical activity | 269 (63.29) | 660 (72.37) | -0.20 |
| ***Disease history*** |  |  |  |
| Hypertension | 76 (57.14) | 468 (51.32) | 0.12 |
| Diabetes mellitus | 30 (22.39) | 151 (16.56) | 0.15 |
| Heart disease | 47 (37.01) | 275 (30.15) | 0.15 |
| Hyperlipidemia | 26 (20.63) | 229 (25.11) | -0.10 |
| Gout | 24 (18.32) | 102 (11.18) | 0.22 |
| Hyperuricemia | 19 (14.50) | 95 (10.42) | 0.13 |
| Arthritis | 25 (23.58) | 179 (19.63) | 0.10 |
| Osteoporosis | 23 (18.7) | 162 (17.76) | 0.02 |
| Stroke | 11 (9.02) | 56 (6.14) | 0.12 |
| Cataract | 71 (51.82) | 419 (45.94) | 0.12 |
| Cancer | 8 (1.84) | 52 (5.7) | -0.19 |
| Fall history | 245 (57.65) | 211 (23.14) | 0.73 |
| Sleep impairment | 58 (46.77) | 409 (44.85) | 0.04 |
| Taking sleeping pills | 45 (10.37) | 213 (23.36) | -0.33 |
| ***Frailty status*** |  |  |  |
| Robust (0 components) | 117 (30.08) | 369 (40.46) | -0.21 |
| Pre-frailty (1~2 components) | 195 (50.13) | 443 (48.57) | 0.03 |
| Frailty (≥ 3 component) | 77 (19.79) | 100 (10.96) | 0.26 |
| ***Frailty components*** |  |  |  |
| Shrinking | 44 (10.68) | 119 (13.05) | -0.07 |
| Poor endurance and energy | 30 (7.13) | 37 (4.06) | 0.14 |
| Low physical activity | 129 (30.79) | 165 (18.09) | 0.31 |
| Slowness | 218 (51.05) | 329 (36.07) | 0.30 |
| Weakness | 167 (41.34) | 273 (29.93) | 0.24 |

MMSE: Mini–Mental State Examination.
